# Conducting Meta-Analyses Based on *p* Values: Reservations and Recommendations for Applying *p*-Uniform and *p*-Curve

**DOI:** 10.1177/1745691616650874

**Published:** 2016-09-29

**Authors:** Robbie C. M. van Aert, Jelte M. Wicherts, Marcel A. L. M. van Assen

**Affiliations:** 1 Department of Methodology and Statistics, Tilburg University; 2 Department of Social and Behavioral Sciences, Utrecht University

**Keywords:** *p*-uniform, *p*-curve, meta-analysis, *p*-hacking, heterogeneity

## Abstract

Because of overwhelming evidence of publication bias in psychology, techniques to correct meta-analytic estimates for such bias are greatly needed. The methodology on which the *p*-uniform and *p*-curve methods are based has great promise for providing accurate meta-analytic estimates in the presence of publication bias. However, in this article, we show that in some situations, *p*-curve behaves erratically, whereas *p*-uniform may yield implausible estimates of negative effect size. Moreover, we show that (and explain why) *p*-curve and *p*-uniform result in overestimation of effect size under moderate-to-large heterogeneity and may yield unpredictable bias when researchers employ *p*-hacking. We offer hands-on recommendations on applying and interpreting results of meta-analyses in general and *p*-uniform and *p*-curve in particular. Both methods as well as traditional methods are applied to a meta-analysis on the effect of weight on judgments of importance. We offer guidance for applying *p*-uniform or *p*-curve using R and a user-friendly web application for applying *p*-uniform.

Meta-analysis is the standard technique to synthesize effect sizes of several studies on the same phenomenon. A well-known problem of meta-analysis is that effect size can be overestimated because of *publication bias* (e.g., Ioannidis, 2008; Lane & Dunlap, 1978). In this article, we define publication bias as the tendency for studies with statistically significant results to be published at a higher rate than studies with results that are not statistically significant. Because evidence of publication bias is overwhelming across many scientific disciplines (Fanelli, 2012), it is important to develop techniques that correct the meta-analytic estimate for publication bias (Moreno, Sutton, Ades, et al., 2009). Recently, van Assen, van Aert, and Wicherts (2015) and Simonsohn, Nelson, and Simmons (2014a) independently developed methods aiming to provide an accurate meta-analytic estimate in the presence of publication bias. Both of their methods, *p*-uniform and *p*-curve, respectively, make use of the distribution of statistically significant results yet differ in implementation. Our goals in this article are to introduce and explain both methods and their differences, to provide straightforward recommendations applying to meta-analysis, and to formulate guidelines for applying and interpreting results of *p*-uniform and *p*-curve.

## A Primer on *p*-Uniform and *p*-Curve

Simonsohn, Nelson, and Simmons (2014b) described how statistically significant *p* values of studies on an effect could be used to test this effect against the null hypothesis that the effect equals zero. This idea was not new; Fisher (1925) developed a method for testing the null hypothesis of no effect by combining *p* values. However, the novelty of *p*-curve lies in its use of only the statistically significant *p* values, which arguably are not affected by publication bias. The method was called *p*-curve because it can be used to analyze the curve or distribution of *p* values. In the logic of the *p*-curve method, there is no effect in the studies in the meta-analysis if the *p* values are uniformly distributed (i.e., *p*-curve is flat), whereas there is an effect if the *p* value distribution or *p*-curve is right skewed (Hung, O’Neill, Bauer, & Köhne, 1997).

A disadvantage of *p*-curve at that time was that effect size could not be estimated. Van Assen et al. (2015) developed another method of analyzing statistically significant *p* values called *p-uniform*, which can be used to estimate the effect size in a set of studies. These researchers called their method *p*-uniform because the effect-size estimate is equal to the value for which the *p* value distribution conditional on that value is uniform (as we explain later). Besides estimating the effect size, *p*-uniform also can be used to estimate a confidence interval (CI) around the effect-size estimate, in addition to testing for publication bias and, similar to *p*-curve (Simonsohn et al., 2014b), for testing the null hypothesis of no effect. Simonsohn et al. (2014a) later extended *p*-curve to estimate effect size as well. However, *p*-curve provides neither a CI nor a test for publication bias. In the present study, we focus on effect-size estimation using both *p*-curve and *p*-uniform.

The strengths of *p*-uniform and *p*-curve and the logic upon which they are based were convincingly illustrated by van Assen et al. (2015) and Simonsohn et al. (2014a). They showed that the methods provide accurate effect-size estimates in the presence of publication bias, even when the number of statistically significant studies is small. Similarly, both methods were found to perform well when studies have the same sample sizes or different sample sizes and when there is (small) heterogeneity of effect size (i.e., when the underlying population effect sizes actually differ between studies in the meta-analysis). Moreover, results of Simonsohn et al. (2014a) suggested that *p*-hacking—or the original researchers’ use of strategies to achieve statistical significance (Simmons, Nelson, & Simonsohn, 2011)— leads to an underestimation of effect size in analyses based on *p*-curve, whereas it leads to overestimation of effect size in traditional meta-analysis (Bakker, van Dijk, & Wicherts, 2012).

## Three reservations

Although we are convinced of the potential and validity of the logic of *p*-uniform and *p*-curve, we have added three important reservations to the application of the methods and the general methodology in its current state. More specifically, we first show that *p*-uniform and *p*-curve may yield implausible negative (*p*-uniform) or inaccurate (*p*-curve) estimates in meta-analyses with *p* values close to the significance level (considered equal to .05 in the present article). Second, we explain why and show that *p*-hacking does not always cause the effect sizes of *p*-curve and *p*-uniform to be underestimated as was stated in Simonsohn et al. (2014a). Finally, we show that in contrast to the results in Simonsohn et al. (2014a), *p*-uniform and *p*-curve cannot deal with a substantial amount of heterogeneity (i.e., there is no single true effect size underlying the studies in the meta-analysis but rather a distribution of true effect sizes). Based on our explanation of the methods and the reservations, we have formulated recommendations for applying meta-analysis in general and interpreting results of *p*-uniform and *p*-curve in particular. These hands-on recommendations are summarized in Table 1. Scientists who consider using these methods should be aware of conditions in which the methods either should not be interpreted or should be interpreted with caution.

**Table 1.** Recommendations for Meta-Analysis and Application of p-Uniform and p-Curve

1. Check for evidence of *p*-hacking in the primary studies. In case of strong evidence or strong indications of *p*-hacking, be reluctant in interpreting estimates of traditional meta-analytic techniques and *p*-uniform and *p*-curve because their effect-size estimates may be biased in any direction depending on the type of *p*-hacking used. 2. Apply fixed-effect and random-effects meta-analysis, as well as *p*-uniform or *p*-curve, and report results conforming to the Meta-Analysis Reporting Standards (MARS; American Psychological Association, 2010, pp. 251–252.). 3. Check for direct or indirect evidence of publication bias. In case of evidence of publication bias, interpret results of *p*-uniform or *p*-curve rather than those of fixed-effect and random-effects meta-analysis; in the absence of such evidence, interpret results of fixed-effect and random-effects meta-analysis. 4. Set the effect-size estimate of *p*-uniform or *p*-curve equal to zero if the average *p* value of the statistically significant studies is larger than .025 5a. If the effect size is homogeneous or if the heterogeneity is small to moderate (*I*^2^ < 0.5), interpret the estimates of *p*-uniform and *p*-curve as estimates of the average population effect size; otherwise, these methods result in overestimates of average population effect size and should be interpreted as estimates of the average true effect size of only the set of statistically significant studies. 5b. In case of substantial heterogeneity (and if desired), create homogeneous subgroups of primary studies on the basis of theoretical or methodological considerations to estimate with *p*-uniform and *p*-curve the average population effect size underlying the studies in each subgroup

In the remainder of the article, we illustrate major issues involved in applying *p*-curve and *p*-uniform by considering a recent meta-analysis of studies on the effect of weight on judgment of importance (Rabelo, Keller, Pilati, & Wicherts, 2015). We briefly describe other methods of meta-analysis using statistically significant effect sizes, introduce the basic idea underlying *p*-uniform and *p*-curve, and illustrate the logic of and computations in *p*-uniform and *p*-curve in Appendix A. The analyses that form the basis of our three reservations and recommendations are presented in the next sections. Readers who do not want to delve into the (technical) details of *p*-uniform and *p*-curve can skip these sections and move over to the Discussion and Conclusion section, where we explain the recommendations in Table 1. R code for all our analyses is available in the Supplemental Materials.

## Example

Rabelo et al. (2015) conducted a meta-analysis on the effect of weight on judgments of importance. The theory underlying the studies included in the meta-analysis is that the physical experience of weight (e.g., holding a heavy object) influences how much importance people assign to things, issues, and people (IJzerman, Padiotis, & Koole, 2013; Jostmann, Lakens, & Schubert, 2009). For instance, in their second study, Jostmann et al. (2009) found that participants who held a heavy clipboard attributed more importance to fairness in decision making than did participants holding a light clipboard. Table B1 in the Appendix B provides the full references, sample sizes ($n_{i}^{1}$ and $n_{i}^{2}$), *t* values, and *p* values from the 25 studies of this kind published in the embodiment literature.

According to the first recommendation, we should consider the presence of *p*-hacking in the primary studies included in the meta-analysis. We believe that the studies on the link between weight and importance are mostly studies in which the specifics of the analysis often are neither preregistered nor clearly restricted by theory. Hence, according to Recommendation 1, we would use caution in interpreting the current results and await new (preferably preregistered) studies in this field.

Four different meta-analytic estimates of the (mean) effect size underlying the weight-importance studies are presented in Table 2. In line with Recommendation 2, we first fitted traditional fixed-effect and random-effects meta-analysis. Both analyses yielded the same effect size estimate of 0.571 (95% CI: [0.468, 0.673]), which is highly statistically significant (*z* = 10.90, *p* < .001) and suggests a medium-to-large effect of the experience of weight on how much importance people assign to things (see Table 2). The results of *p*-uniform’s publication bias test suggested evidence of publication bias (*z* = 5.058, *p* < .001), so the results of *p*-uniform or *p*-curve should be interpreted rather than the standard meta-analytic estimates (Recommendation 3). Because the average *p* value of the 23 statistically significant studies equaled .0281, we set the effect size estimate of *p*-uniform and *p*-curve equal to zero, in line with Recommendation 4. When the estimate is not set to zero, application of *p*-curve and *p*-uniform yields a nonsignificant negative effect size (see Table 2), and the 95% CI for *p*-uniform ([−0.676, 0.160]) suggests that the effect size is small at best.

**Table 2.** Results of p-Uniform, p-Curve, Fixed-Effect Meta-Analysis, and Random-Effects Meta-Analysis Applied to the Meta-Analysis Reported in Rabelo, Keller, Pilati, and Wicherts (2015)

			Fixed-effect	Random-effects
Effect-size estimate	−0.179	−0.172	0.571	0.571
95% CI	[−0.676, 0.160]		[0.468, 0.673]	[0.468, 0.673]
Test of H_0_: δ = 0	*z* = 0.959, *p* = .831	χ^2^(46) = 55.833, *p* = .848	*z* = 10.904, *p* < .001	*z* = 10.904, *p* < .001
Publication bias test	*z* = 5.058, *p* < .001			

*Note*. H_0_: δ = 0 refers to the null hypothesis of no effect. CI = confidence interval.

The null hypothesis of no heterogeneity among the included studies was not rejected, *Q*(24) = 4.55, *p* = 1, *I*^2^ = 0, which suggests that *p*-uniform and *p*-curve may accurately estimate the average population effect size (Recommendation 5a). Note that due to the absence of heterogeneity, effect-size estimates of fixed-effect and random-effects meta-analysis were identical. Although the lack of heterogeneity suggests that the effects are homogeneous, in this particular instance, homogeneity is excessive (with a *p* value of the *Q* test very close to 1). Such excessive homogeneity is unlikely to occur under normal sampling conditions (Ioannidis, Trikalinos, & Zintzaras, 2006) and could be caused by publication bias (Augusteijn, 2015), possibly in combination with *p*-hacking. Our preliminary conclusion about the effect of physical experience of weight on importance would be that there is as yet no evidence in the literature for such an effect.

## Other Methods Using *p* Values for Estimation

Several other methods were developed in which *p* values are used to obtain an effect-size estimate corrected for publication bias. Hedges (1984) developed a method for correcting meta-analytic effect sizes for publication bias that is similar to *p*-uniform and *p*-curve. He derived the maximum likelihood estimator of effect size under a model with only statistically significant results and studied the bias in the effect-size estimate. Although Hedges (1984) discussed the application to meta-analyses, he only examined the bias in effect size of one statistically significant study. Hedges’s method and its performance are not further examined in this article because it is currently not applied in practice.

Other methods for obtaining effect-size estimates corrected for publication bias are selection models (Hedges & Vevea, 2005). Selection models use an effect-size model and a weight function for correcting the effect-size estimates for publication bias. The effect-size model describes the distribution of effect sizes in case all studies are published. The weight function yields probabilities of observing a particular study given its effect size or *p* value.

Effect sizes of the studies then are weighted by these probabilities in order to obtain an effect size corrected for publication bias (for an overview on selection models, see Hedges & Vevea, 2005). Drawbacks of selection models are that they require a large number of studies (i.e., more than 100) in order to avoid nonconvergence (e.g., Field & Gillett, 2010; Hedges & Vevea, 2005), often yield implausible weight functions (Hedges & Vevea, 2005), are hard to implement, and require sophisticated assumptions and difficult choices (Borenstein, Hedges, Higgins, & Rothstein, 2009, p. 281). A recently proposed alternative for selection models based on Bayesian statistics showed promising results and does not have convergence problems when the number of studies in the meta-analysis is small (Guan & Vandekerckhove, 2015). However, a disadvantage of the latter method is that it makes stronger assumptions on weight functions than *p*-uniform and *p*-curve. In *p*-uniform and *p*-curve, the probability of publishing a finding is assumed to be independent of its *p* value given its statistical significance, whereas the models in the method described in Guan and Vandekerckhove (2015) assume specific weights of findings depending on their *p* value, significant or not. Because both significant and nonsignificant *p* values are included, this Bayesian method makes assumptions about the extent of publication bias, and its estimates are affected by the extent of publication bias. For these reasons, we will not discuss selection models and their properties further.

## Basic Idea Underlying *p*-Uniform and *p*-Curve

In both *p*-uniform and *p*-curve, the distribution of only the statistically significant *p* values are used for estimating effect size for at least two reasons. First, collecting unpublished studies without the existence of study (or trial) registers is often hard, and these unpublished studies may provide biased information on effect size just as published studies do (Ferguson & Brannick, 2012). Second, evidence for publication bias is overwhelming. For instance, researchers have estimated that at least 90% of the published literature within psychology contains statistically significant results (e.g., Bakker et al., 2012; Fanelli, 2012; Sterling, Rosenbaum, & Weinkam, 1995), yielding overestimated effect sizes (e.g., Ioannidis, 2008; Lane & Dunlap, 1978). Because most published findings are statistically significant, only a relatively small number of published but statistically nonsignificant studies (on average up to 10%) need to be omitted from meta-analyses by *p*-curve and *p*-uniform.

Both *p*-uniform and *p*-curve are founded on the statistical principle that the distribution of *p* values conditional on the true effect size is uniform.^1^ This same statistical principle underlies standard null-hypothesis significance testing, where the *p* values are uniformly distributed when the true effect size equals zero. In contrast to null-hypothesis significance testing, *p* values from *p*-uniform and *p*-curve are computed not only conditional on an effect size of zero (which would yield a simple transformation of the traditional *p* values) but also conditional on other effect sizes (in which case the conditional *p* value is not a simple transformation of the traditional *p* value anymore). The effect-size estimate of *p*-uniform and *p*-curve represents the effect size for which the conditional *p* values are uniformly distributed.^2^ What both procedures do is to find an underlying effect, compute for each study the (conditional) *p* value given this effect, and subsequently check whether these conditional *p* values show a flat (i.e., uniform) distribution, which they should if indeed the studies reflect that underlying effect. The assumptions of *p*-uniform and *p*-curve are that all statistically significant studies have the same probability of getting published and being included in the meta-analysis and are statistically independent (i.e., they should not be based on the same sample, van Assen et al., 2015). We describe the logic underlying *p*-uniform and *p*-curve as well as how the conditional *p* value and effect-size estimate for *p*-uniform and *p*-curve are computed in Appendix A.

## If Heterogeneity Is Moderate to Large, *p*-Curve and *p*-Uniform Overestimate Effect Size

Simonsohn et al. (2014a) stated that *p*-curve provides accurate effect-size estimates in the presence of heterogeneity (i.e., in cases where true effects underlying the observed effects of the studies differ). In a blog post, Simonsohn (2015) qualified this statement as follows: “If we apply *p*-curve to a set of studies, it tells us what effect we expect to get if we run those studies again.” In other words, applying *p*-curve (and *p*-uniform) to a set of studies yields an accurate estimate of the average true effect size of *this exact set of studies*. However, we note that it may be impossible to run exactly the same studies again since there will always be differences in, for instance, the participants included in the studies and the context in which the studies were conducted.

Because of the importance of its implications for the interpretation of the *p*-curve’s estimate, we provide a simple example with heterogeneous effect sizes. Assume that the true effect size is equal to either 0 or 1 and that both underlying effects are equally likely, which implies an average true effect size, µ = .5. Also assume that both true effect sizes are investigated with the same number of studies with a huge sample size, implying 5% and 100% of studies with true effects equal to 0 and 1 are statistically significant, respectively. Because the sample sizes of the studies are huge, the observed effect sizes of statistically significant studies are equal to (a number very close to) 0 and 1. As a result, the *p*-curve’s estimate equals (0.05 × 0 + 1 × 1)/1.05 = .952, which is indeed equal to the average underlying true effect size of all the statistically significant studies. However, it is much larger than the true population average of .5. Moreover, traditional random-effects meta-analysis provide a more accurate estimate of true average effect size (i.e., less positively biased) than *p*-curve, even under extreme publication bias.

It is often unrealistic to assume homogeneous true effect sizes underlying primary studies in psychological meta-analyses (e.g., Borenstein et al., 2009). Moreover, researchers often want to estimate the true effect size in the population instead of the average true effect size in the studies included in the meta-analysis. That is, meta-analysts wish to obtain an estimate of .5, rather than .952 in our example. The reason that *p*-curve overestimates effect size under heterogeneity is that studies with an underlying true effect of zero have a lower probability of being statistically significant, such that these studies are underrepresented in the meta-analysis. In our example, studies with large true effect size are 20 times more likely to be included in the meta-analysis than those with a zero effect size. Finally, we note that in this simple example, we could deal with the heterogeneity rather easily if true effect size (0 or 1) is perfectly linked to an observed dichotomous study characteristic; applying *p*-curve or *p*-uniform to studies of both groups (a so-called *subgroup analysis*, e.g., Borenstein et al., 2009) yields the correct estimates of 0 and 1. We therefore recommend applying these methods to subgroups of studies on the basis of the different levels of a moderator in order to create more homogeneous sets of studies (Recommendation 5b). However, in other realistic situations, the causes of heterogeneity are not simply observed, and subgroup analysis will not completely solve the heterogeneity problem.

To illustrate the effect of heterogeneity of effect sizes on the (over)estimation of effect size by *p*-curve and *p*-uniform, we also performed a simulation study in which we varied heterogeneity from moderate to large under the usual scenario: heterogeneity was modeled continuously using a normal distribution of true effects, which is commonly assumed in meta-analysis (Raudenbush, 2009). As in Simonsohn et al. (2014a), 5,000 studies with statistically significant results were generated on which the meta-analysis was conducted. All studies had two conditions with 50 cases each, with population variance equal to 1 in both conditions. Average population effect size was .397, and standard deviations of true effect size (denoted by τ) were 0, 0.2, 0.4, 0.6, and 1, roughly corresponding to *I*^2^ (i.e., ratio of heterogeneity to total variance; Higgins & Thompson, 2002) values of 0, .5 (moderate heterogeneity), .8 (large heterogeneity), .9, and .96 in the population of studies. Table 3 provides the estimates of *p*-curve, *p*-uniform, fixed-effect meta-analysis, and random-effects meta-analysis (with restricted maximum likelihood estimator for estimating the amount of heterogeneity) of all studies with a statistically significant positive effect. For *p*-uniform, we used the Irwin-Hall estimator and the so-called “1 − *p*” estimator, a variant based on Fisher’s method, because this estimator is least affected by extreme effect sizes and therefore provides better estimates in case of heterogeneity (van Assen et al., 2015).

**Table 3.** Estimates of Effect Size for 5,000 Studies With Statistically Significant Positive Effects

Method	*τ* = 0, *I*^2^ = 0	*τ* = .2, *I*^2^ = .5	*τ* = .4, *I*^2^ = .8	*τ* = .6, *I*^2^ = .9	*τ* = 1, *I*^2^ = .96
*p*-curve	.393	.530	.703	.856	1.094
*p*-uniform					
Irwin-Hall estimator	.383	.535	.724	.874	1.110
1 − *p* estimator	.387	.522	.679	.776	.903
Fixed-effect	.553	.616	.738	.875	1.104
Random-effects	.553	.616	.743	.897	1.185

*Note*. Fixed-effect and random-effects meta-analysis performed with restricted maximum likelihood for estimating the amount of heterogeneity under different levels of heterogeneity (true effect .397).

The first column confirms that *p*-curve and *p*-uniform provide accurate estimates under homogeneity (effect-size estimates are close to the true effect size of .397), whereas fixed-effect and random-effects meta-analysis (both .553) overestimate effect size. The other columns, however, show that both *p*-curve and *p*-uniform overestimate the mean population effect size of .397 for moderate-to-large heterogeneity and that this bias increases with larger heterogeneity. Note that the bias of fixed-effect and random-effects meta-analysis also increases with larger heterogeneity and exceeds the bias of *p*-curve and *p*-uniform in these cases. Although the 1 − *p* estimator of *p*-uniform provides the best estimates, its bias is still so large that we do not recommend applying the methodology in its current state to estimate the average population effect size in situations where moderate or large heterogeneity is present or suspected (Recommendation 5a).

**Fig. 1.**
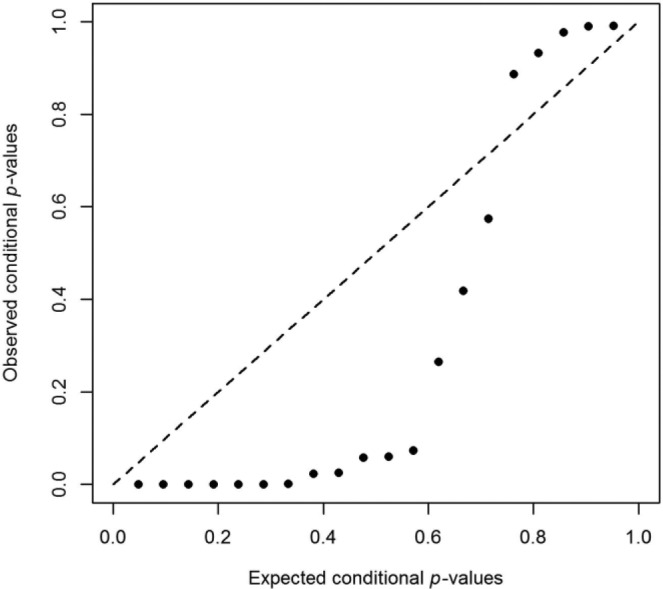
Probability-probability (P-P) plot for a meta-analysis of 20 studies with large heterogeneity.

For illustrative purposes, we show how *p*-curve and *p*-uniform could still be used to diagnose heterogeneity by applying *p*-uniform to one simulated meta-analysis of 20 studies with the aforementioned specifications; mean population effect size equal to .397, and large heterogeneity (τ = 1; *I*^2^ = .96). The 1 − *p* estimator of *p*-uniform yielded an effect size estimate of $\hat{\delta }$ = .795. However, a comparison of the expected conditional *p* values with the observed conditional *p* values for $\hat{\delta }$ = .795 in the probability-probability (or P-P) plot in Figure 1 clearly indicated systematic misfit. Specifically, observed conditional *p* values should be uniformly distributed, as the expected conditional *p* values. That is, all dots should fall on or close to the diagonal. However, assuming a fixed effect size of .795, the observed conditional *p* values were either (much) too small (dots below the diagonal to the left) or (much) too large (dots above the diagonal to the right), signifying a large effect size variance. In other words, deviations from the diagonal in the P-P plot may be used to diagnose heterogeneity of effect size.

To conclude, if moderate to large heterogeneity is present, then *p*-curve and *p*-uniform estimate the average true effect underlying all significant studies in the meta-analysis. If the main goal of the meta-analysis is to estimate the average true effect of the whole population of studies in the presence of heterogeneity (*I*^2^ ≥ .5), we do not recommend using *p*-curve or *p*-uniform because doing so generally overestimates average true effect size (Recommendation 5a). In opposition to mainstream meta-analytic thinking, Simonsohn et al. (2014a) argued that “the” average true effect size under heterogeneity often does not exist and even that it is meaningless because studies cannot be run randomly. However, we believe the average true effect size may be interpreted meaningfully in the presence of heterogeneity in some situations and consider heterogeneity to be both realistic for psychological studies (e.g., in 50% of the replicated psychological studies in the Many Labs Replication Project, heterogeneity was present; Klein et al., 2014) and important to take into consideration when estimating average effect size.

## Sensitivity to *p* Values Close to .05

Statistically significant *p* values that are uniformly distributed in [0, .05] are in line with a zero true effect size. A distribution of *p* values with many *p* values close to .05 (and, say, an average *p* value of more than .025) is not in line with a zero true effect size but may indicate a negative true effect size. We now show that if most of studies in the meta-analysis have a *p* value just below the significance criterion of .05, then *p*-uniform yields implausible highly negative effect size estimates and a very wide CI. Similarly, under these conditions *p*-curve behaves erratically.

To illustrate the consequences of having many *p* values just below .05 on the estimates of *p*-uniform and *p*-curve, consider doing a meta-analysis on the following three observed effect sizes with two conditions having equal sample sizes: Effect 1 with *d* = 0.963, *t*(18) = 2.154, *p* = .045 (two-tailed); Effect 2 with *d* = 0.582, *t*(48) = 2.058, *p* = .045; and Effect 3 with *d* = 0.4, *t*(98) = 2.002, *p* = .048. Several explanations exist for observing multiple *p* values that barely pass the significance criterion as in this example. First, *p*-hacking such as optional stopping or data peeking (Hartgerink, van Aert, Nuijten, Wicherts, & van Assen, 2015; Lakens, 2014) or the deletion of outliers to achieve statistical significance may yield a preponderance of *p* values just below .05 (Bakker & Wicherts, 2014b). Another explanation is (bad) luck—when the meta-analysis consists of a small number of studies, and multiple studies coincidentally have *p* values close to .05. The fixed-effect meta-analytic estimate for these three observed effect sizes is .506 (*p* <.001), with a 95% CI excluding zero [.199, .812].^3^

**Fig. 2.**
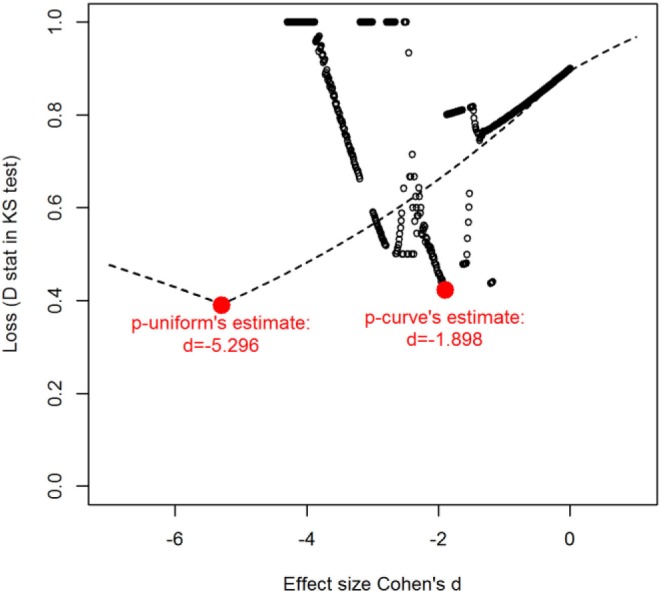
Values for Kolmogorov-Smirnov (KS) test statistics in implementation of *p*-curve and *p*-uniform for the example with three observed effect sizes and *p* values close to .05. D stat = test statistics of KS test.

Applying *p*-curve to this set of studies yields an effect size estimate of *d* = −1.898. Figure 2 displays the behavior of the Kolmogorov-Smirnov test statistic in *p*-curve with dots as a function of effect size. It shows that the Kolmogorov-Smirnov statistic in *p*-curve does not behave as it should (decrease to one minimum, and then increase, and be continuous for all effect sizes). This erratic behavior is caused by implementation of *p*-curve using the *t* distribution from the software R (R Core Team, 2015), because R yields inaccurate probabilities for very high *t* values in combination with an extreme noncentrality parameter (Witkovský, 2013). This inaccuracy may cause conditional *p* values to be negative or undefined (division by zero), which yields the discontinuities in Figure 2. Therefore, *p*-curve’s estimate cannot be trusted for this example.

The implementation of *p*-uniform differs from that of *p*-curve because the normal distribution (instead of the *t*-distribution) is used for computing conditional *p* values. The studies’ effect sizes are transformed into standardized effect sizes (Hedges’ *g*) before the effect size is estimated. Consequently, extreme tail probabilities can be computed, and therefore *p*-uniform behaves as it should, as can be seen from the dashed line in Figure 2. At the same time, the *p*-uniform’s estimate, also based on the Kolmogorov-Smirnov statistic to ease comparison with *p*-curve, is −5.296, which is clearly peculiar. Because a confidence interval cannot be computed with the Kolmogorov-Smirnov statistic, we also calculated the Irwin-Hall estimates with *p*-uniform; $\hat{\delta }$ = −5.484, 95% CI [−15.219, −1.009]. Although the behavior of the *p*-uniform estimator is correct, its effect size estimate (< −5) is unrealistically low; the probability of obtaining three positive statistically significant studies when δ = −5.484 is essentially zero. Furthermore, the CI of *p*-uniform is very wide. We explain in the Supplemental Materials why these implausible negative estimates can be obtained and what can be concluded from these estimates. To deal with the implausibly negative estimates of *p*-uniform and the erratic behavior of *p*-curve, we recommend setting the effect-size estimate of *p*-uniform and *p*-curve to zero in meta-analyses where the mean of the significant *p* values of the primary studies is larger than .025 (Recommendation 4). The cutoff of .025 is natural for two reasons. First, if the average *p* value equals .025, *p*-uniform actually estimates $\hat{\delta }$ = 0. Second, average *p* values higher than .025 yield negative effect size estimates, making testing redundant because the *p* value of the test would be above .5 and hence could not be statistically significant. Of course, the true effect size can be below zero, but a left-tailed hypothesis test then is required to examine whether the effect is smaller than zero.

## Bias in Effect Size Estimates for *p*-Uniform and *p*-Curve From *p*-Hacking

Simonsohn et al. (2014a) examined the effect of *p*-hacking on effect-size estimation in *p*-uniform, considering three different *p*-hacking strategies: data peeking, selectively reporting using three dependent variables, and selectively excluding outliers. In data peeking (or optional stopping), observations are added whenever a test is not yet statistically significant. Their *p-*hacking strategy with multiple dependent variables refers to a practice whereby dependent variables are considered one by one, until one is found for which the test was statistically significant, which is then published. Selectively excluding outliers refers to deleting outliers whenever a test is not yet statistically significant. From their simulations of specific examples of these three practices, they concluded that *p*-curve underestimates effect sizes. However, *p*-hacking comprises a large number of behaviors, and Simonsohn et al. (2014a) examined only three of these behaviors. We now show that other types of *p*-hacking lead to overestimation of effect size in *p*-curve and *p*-uniform.

As Simonsohn et al. (2014a, p. 670) explained, *p*-hacking affects the *p*-curve’s estimate through the conditional *p* value distribution. For instance, data peeking and selectively excluding outliers lead to a distribution with relatively more conditional *p* values corresponding to just statistically significant results, which pulls the *p*-curve (and the *p*-uniform) estimate downward, as we explained in the foregoing section. On the other hand, *p*-hacking that yields relatively more small *p* values results in an overestimation of effect size. Ulrich and Miller (2015) and Bruns and Ioannidis (2016) illustrated that multiple *p*-hacking behaviors may result in relatively more small *p* values, which leads to overestimation of effect size in *p*-curve (and *p*-uniform).

We examined the effect of two methods of *p*-hacking on effect-size estimation in *p*-curve and *p*-uniform. The first method again involves selectively reporting among three dependent variables but differs from the procedure in Simonsohn et al. (2014a) in one crucial aspect: Instead of the *first* significant *p* value, the *smallest* of three significant *p* values is reported. The second method involves a “multiple-conditions” scenario, whereby multiple experimental conditions are tested and compared with the same control condition, and only the comparison yielding the largest difference (and smallest *p* value) is reported. We note that a large portion of surveyed psychologists have admitted to using at least once selective reporting among different dependent variables (63.4%) and not reporting all experimental conditions (27.7%) in their work (John, Loewenstein, & Prelec, 2012).

**Fig. 3.**
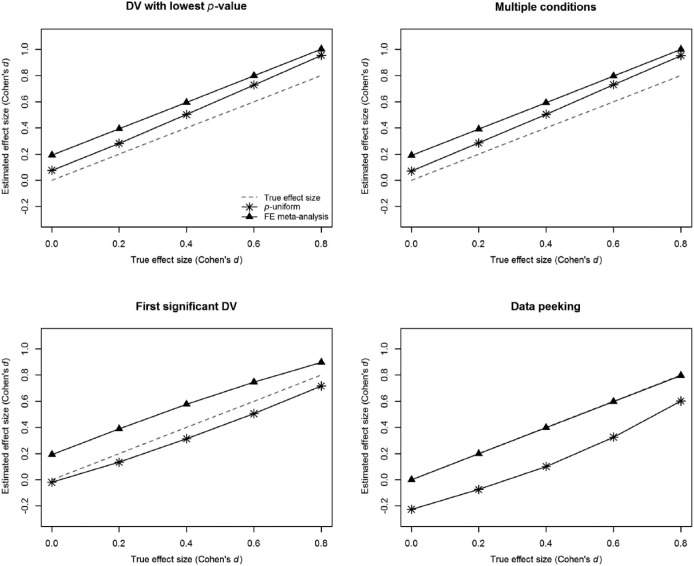
Effect size estimates in *p*-uniform and fixed-effect meta-analysis in case of four types of *p*-hacking. FE = fixed-effect; DV = dependent variable.

Figure 3 presents the estimates of *p*-uniform, as well as the true effect size and the effect size of fixed-effect meta-analysis (see the Supplemental Materials for the details of our simulations). We do not show the *p*-curve results because these are almost indistinguishable from the *p*-uniform results. Conditions of “first significant dependent variable (DV)” and “data peeking” replicate the simulations in Simonsohn et al. (2014a), showing that *p*-uniform and *p*-curve indeed underestimate effect size under these conditions. The estimate is slightly below the true effect size for the “first significant DV” and about .2 lower on the scale of Cohen’s *d* for “data peeking” for all true effect sizes from 0 (*no effect*) to .8 (*considered a large effect*). Conversely, and as anticipated, both “DV with lowest *p* value” and “multiple conditions” overestimate effect size, and this overestimation increases for larger true effect sizes. What should also be mentioned is that *p*-uniform and *p*-curve did not always outperform traditional fixed-effect meta-analysis in the *p*-hacking scenarios we simulated. For instance, fixed-effect meta-analysis outperformed *p*-uniform and *p*-curve (i.e., presented less-biased estimates) in the case of data peeking (e.g., Francis, 2013; van Aert, Maassen, Wicherts, & van Assen, 2016). We therefore concluded that (a) *p*-hacking may bias *p*-uniform and *p*-curve estimates in any direction depending on the type of *p*-hacking and (b) *p*-uniform and *p*-curve estimates are not necessarily better than those of fixed-effect meta-analysis when *p*-hacking occurs. Thus, *p*-uniform and *p*-curve can deal with publication bias, but (just like traditional fixed-effect and random-effects meta-analysis) neither method corrects for *p*-hacking or reacts predictably to it.

Because the validity of results of both traditional meta-analytic methods and *p*-curve and *p*-uniform may be lowered by *p*-hacking, we recommend scrutinizing both data and studies included in the meta-analysis before applying meta-analytic methods. Underpowered primary studies (i.e., statistical power substantially below 0.8) and a preponderance of *p* values just below .05 are signals for *p*-hacking. Other signals are unsystematic deletion of outliers and reporting results of other than commonly used measurement instruments. If there are signs of *p*-hacking, we recommend that applied researchers be reluctant to interpret the results of any meta-analysis (Recommendation 1).

## Discussion and Conclusion

Recently, new methods were developed to provide an accurate meta-analytic estimate in the presence of publication bias (Simonsohn et al., 2014a; van Assen et al., 2015). These methods, *p*-uniform and *p*-curve, are based on the same basic idea but differ in implementation. The idea underlying these methods is to select only the statistically significant results and estimate the effect size using the principle of statistical theory that the distribution of (conditional) *p* values based on the true effect size is uniform. The researchers van Assen et al. (2015) and Simonsohn et al. (2014a) convincingly demonstrated the power of *p*-uniform and *p*-curve and the principles upon which the methods are based to carry out meta-analyses. In this article, we explained the rationale and basics of both methods, added three reservations (concerning heterogeneity, incredible estimates, and *p*-hacking) to the application of both methods, and offered hands-on recommendations for researchers.

We explained that *p*-curve behaves erratically and yields inaccurate estimates in situations in which multiple studies in a meta-analysis have *p* values close to .05. Due to a difference in implementation, *p*-uniform does not exhibit this erratic behavior but provides implausible negative estimates. These problems are solved by setting the estimate in *p*-uniform and *p*-curve to zero whenever the mean of *p* values in statistically significant studies exceeds .025 (i.e., whenever the *p*-uniform estimate is lower than zero). We also showed that *p*-hacking may bias the estimates of *p*-uniform and *p*-curve in any direction depending on the particular type of *p*-hacking, and estimates from these methods are not necessarily better than those of fixed-effect meta-analysis when *p*-hacking has taken place. Finally, we explained that *p*-curve and *p*-uniform estimate the average true effect underlying all significant studies in the meta-analysis but overestimate the average true effect of the whole population of studies whenever moderate-to-large heterogeneity is present.

On the basis of these and contemporary insights, we formulated the recommendations summarized in Table 1. These recommendations hold for any meta-analysis and extend the Meta-Analysis Reporting Standards (MARS) proposed by the American Psychological Association (2010, p. 251–252).

First, we recommend that researchers be reluctant to interpret the results of any meta-analytic technique if there are indicators of *p*-hacking in the primary studies (Recommendation 1) because *p*-hacking may bias the effect-size estimates of meta-analysis in any direction. Indicators of potential *p*-hacking include the unsystematic deletion of outliers in many primary studies, the usage and reporting of multiple and different measures for the same dependent variable across primary studies, the common use of small underpowered studies, inconsistencies between sample size descriptions and degrees of freedom (Bakker & Wicherts, 2014a), and grossly misreported *p* values (Nuijten, Hartgerink, van Assen, Epskamp, & Wicherts, 2015). In addition, *p*-hacking can be characteristic of a particular research field (e.g., different measures of dependent variables in a research field) as well as of a single study or a set of studies. Researchers can conduct a sensitivity analysis by comparing the results of traditional meta-analysis methods and *p*-uniform and *p*-curve with the results of these methods applied to only the studies in which no *p*-hacking is suspected (e.g. because they involved the use of preregistered data collection and analysis plans). Meta-analysts will probably observe indicators of *p*-hacking (if these are present) during the literature search and data extraction and do not have to go through all the primary studies again to gather information about the potential presence of *p*-hacking.

Second, we recommend applying fixed-effect and random-effects meta-analysis and *p*-uniform or *p*-curve (Recommendation 2). The selection of a fixed-effect or random-effects meta-analysis should be based on whether a researcher wants to draw inferences on only the studies included in the meta-analysis (fixed-effect) or wants to generalize the meta-analytic results to the whole population of studies (random-effects; see Borenstein et al., 2009, and Schmidt, Oh, & Hayes, 2009, for a more elaborate discussion on selecting fixed-effect or random-effects meta-analysis). Moreover, the estimate of fixed-effect meta-analysis, compared with the estimate of random-effects meta-analysis, may signal publication bias; publication bias generally results in higher estimates of random effects than fixed-effect meta-analysis because the studies with smaller sample sizes and (usually) overestimated effect sizes get less weight in fixed-effect meta-analysis (Greenhouse & Iyengar, 2009).

Next, we recommend checking for direct and indirect evidence of publication bias (Recommendation 3). Direct evidence can be obtained using *p*-uniform’s publication bias test. Previous research has suggested that *p*-uniform’s publication bias test has higher statistical power than traditional tests (van Assen et al., 2015), which are known to have low statistical power (e.g., Borenstein et al., 2009; Sterne & Egger, 2005). Moreover, use of the quite popular trim-and-fill method is discouraged because it often provides inaccurate results (Moreno, Sutton, Abrams, et al., 2009; Simonsohn et al., 2014a; Stanley & Doucouliagos, 2014; van Assen et al., 2015). However, for a small number of studies in the meta-analysis or a small amount of publication bias, *p*-uniform’s publication bias test lacks sufficient statistical power. In these cases, indirect evidence of publication bias may be used. An example of indirect evidence is if 80% or more of the effect sizes in the primary studies are statistically significant when at the same time the sample sizes of the studies imply a power of .5 or less to detect a medium effect size (e.g., see Francis, 2013). In case of (direct or indirect) evidence of publication bias, we recommend that conclusions be based on the results of *p*-uniform or *p*-curve, rather than on fixed-effect and random-effects meta-analysis, because these traditional methods overestimate effect size in the presence of publication bias (e.g., Bakker et al., 2012; Ioannidis, 2008; Lane & Dunlap, 1978; van Assen et al., 2015). Although *p*-uniform and *p*-curve also provide accurate effect-size estimates even in the absence of publication bias (Simonsohn et al., 2014a; van Assen et al., 2015), we recommend interpreting fixed-effect and random-effects meta-analysis in this case because these traditional methods yield more efficient and precise estimates.

We recommend setting the estimates of *p*-uniform and *p*-curve to 0 if the average *p* value of statistically significant studies is larger than .025 (Recommendation 4); an average larger than .025 signals no evidence of an effect or the use of *p*-hacking in the set of included studies (in which case, effect-size estimation from meta-analytic methods may be biased in any direction depending on the type of *p*-hacking; see Recommendation 1). Interpreting the estimates of *p*-uniform and *p*-curve as the average population effect size estimate is discouraged if the effect-size heterogeneity is large (Recommendation 5a). In this case, the *p*-uniform and *p*-curve estimate reflects the average true effect underlying all significant studies in the meta-analysis. The average population effect size is overestimated (although the addition of *p*-hacking could complicate this pattern further) when there is moderate or large heterogeneity (*I*^2^ ≥ .5), and the average true effect of the whole population of studies is estimated. To deal with heterogeneous effect sizes and still be able to accurately estimate the average true effect of the whole population of studies, one can apply *p*-uniform or *p*-curve to homogeneous subgroups of primary studies created on the basis of theoretical (e.g., same population of participants being studied) or methodological considerations (using the same methodology, i.e. study design and measures; Recommendation 5b). The implication of Recommendations 3 and 5 is that currently no method provides accurate estimates of average population effect size in the presence of both publication bias and heterogeneity.

In the example meta-analysis described earlier, we applied *p*-uniform and *p*-curve to a set of primary studies on the effect of weight on judgment of importance (Rabelo et al., 2015). Researchers can also easily apply *p*-uniform or *p*-curve to their own data. User-friendly R code for applying *p*-uniform can be readily installed.^4^ Moreover, we developed a user-friendly web application for researchers who are not familiar with R (https://rvanaert.shinyapps.io/p-uniform). R code for estimating effect size with *p*-curve can be found in the supplementary materials of Simonsohn et al. (2014a). Advantages that *p*-uniform has over *p*-curve are that *p*-uniform also includes a publication bias test and yields a CI around the effect-size estimate.

To conclude, even though both *p*-uniform and *p*-curve are promising meta-analytic methods, the methodology underlying them is still under development, and properties of these methods still need to be examined under more stringent conditions (e.g., different forms of *p*-hacking). Moreover, both methods need to be extended to allow estimation of other effect sizes, such as odds ratios, which have their own idiosyncrasies. Once the current methodology is refined further—particularly by enabling accurate estimation in case of heterogeneity—we believe it has the potential to become the standard meta-analytic tool correcting for publication bias. At present, however, researchers should follow the recommendations provided in Table 1 to avoid drawing erroneous conclusions from these still-developing methods.

## Figures and Tables

**Fig. A1.**
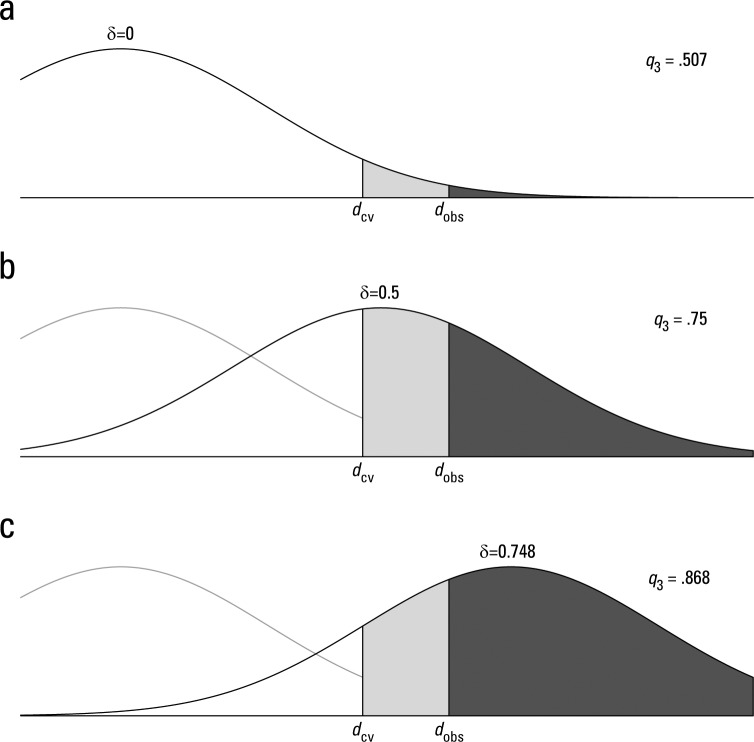
Computation of conditional *p* values for Effect 3 (*q*_3_) for three effect sizes: δ = 0; δ = 0.5 (true effect size); and δ = 0.748 (estimate of fixed-effect meta-analysis). Critical value on Cohen’s *d* scale is denoted by *d*_cv_ and observed effect size is denoted by *d*_obs_.

**Fig. A2.**
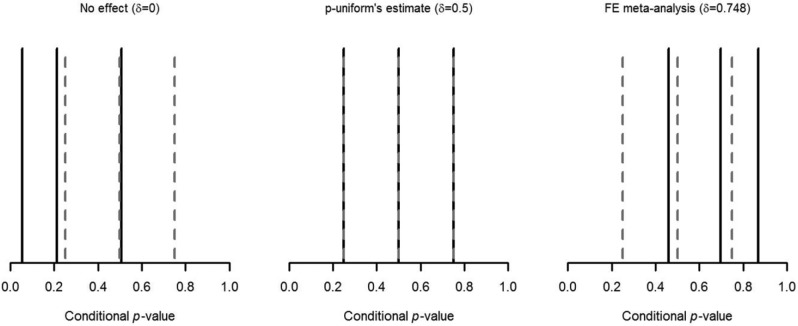
Observed conditional *p* values (solid black lines) and conditional *p* values under uniformity (dashed gray lines) for the example with three observed effect sizes. The three panels refer to the conditional *p* values for the *p*-uniform hypothesis test of no effect (δ = 0), *p*-uniform effect size estimate (δ = 0.5), and effect size obtained by fixed-effect (FE) meta-analysis (δ = 0.748).

**Fig. A3.**
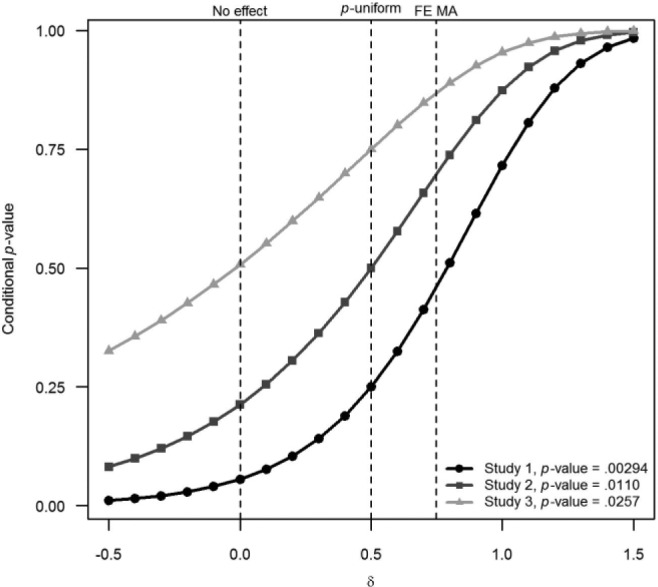
Conditional *p* values as a function of true effect size (*x* axis) for each of the three observed effect sizes in the example. Effect sizes zero (δ = 0), true effect size (δ = 0.5), and estimated by fixed-effect meta-analysis (δ = 0.748) are indicated by vertical lines.

## Appendix A

### Illustration of Logic of and Computations in *p*-Uniform and *p*-Curve

A simple example involving one control condition and one experimental condition with 25 observations each illustrates the logic underlying *p*-uniform and *p*-curve. Imagine that the true effect size (δ) is 0.5 and three statistically significant effects are found with a two-tailed *t* test (α = .05) for testing the null hypothesis of no effect—Effect 1: *d* = 0.872, *t*(48) = 3.133 (two-tailed *p* = .00294); Effect 2: *d* = 0.737, *t*(48) = 2.646 (two-tailed *p* = .0110); and Effect 3: *d* = 0.641, *t*(48) = 2.302 (two-tailed *p* = .0257). Applying traditional fixed-effect meta-analysis to these three observed studies yields an overestimated effect size of 0.748, 95% CI [0.416; 1.080].

Conditional *p* values are used in *p*-curve and *p*-uniform (i.e., conditional on the effect size being statistically significant). More precisely, the conditional *p* value of an observed effect size refers to the probability of observing this effect size or larger, conditional on the observed effect size being statistically significant and given a particular population (or “true”) effect size. Statistical significance has to be taken into account because *p*-uniform and *p*-curve focus only on the interval with *p* values between 0 and .05 rather than on the interval from 0 to 1. Figure A1 depicts how this conditional *p* value of Effect 3 is computed for three different candidates of the underlying effect size, namely, δ = 0, δ = 0.5 (i.e., the true effect size), and δ = 0.748 (i.e., estimate of fixed-effect meta-analysis). Figure A1a reflects the conditional *p* value for δ = 0, which is calculated by dividing the probability of observing an effect size larger than the observed Effect 3 (dark gray area in Figure A1a to the right of observed effect size, *d*_obs_) by the probability of observing an effect size larger than the critical value (light and dark gray areas in Figure A1a to the right of critical value, *d*_cv_). For δ = 0, the null hypothesis being tested, this boils down to dividing the *p* value (.0257) by α = .05, yielding a conditional *p* value (denoted by *q)* for Effect 3 of *q*_3_ = .0257/.05 = .507.^5^ Thus, for δ = 0, the conditional *p* value is simply 20 times the traditional *p* value.

In computation of the conditional *p* values under effects that differ from zero, calculations closely resemble the computation of statistical power of a test. Consider the conditional *p* value of Effect 3 at δ = 0.5 (Figure A1b). The critical value (*d*_cv_) and the observed effect size (*d*_obs_) on the Cohen’s *d* scale remain the same, but the distribution of true effect size (δ) is now shifted to the right. The numerator in computing the conditional *p* value expresses the probability that the observed effect size *d*_obs_ is 0.641 or larger, given δ = 0.5 (dark gray area in Figure A1b to the right of *d*_obs_), which equals 0.314, whereas the denominator expresses the probability that the observed effect size is statistically significant given δ = 0.5 (light and dark gray areas in Figure A1b to the right of *d*_cv_), which equals 0.419 (i.e., the traditional power of the study, given its degrees of freedom and δ = 0.5). This yields a conditional *p* value for Effect 3 at δ = 0.5 of *q*_3_ = 0.314/0.419 = 0.75. The conditional *p* value of Effect 3 at δ = 0.748, as displayed in Figure A1c, can be computed in a similar way: *q*_3_ = 0.644/0.742 = 0.868.

The conditional *p* values of all three observed effect sizes in our example under the three different true effect sizes are presented in Figure A2. The solid black lines in the left panel of Figure A2 shows the conditional *p* values for δ = 0:

*q*_1_ = .00294/.05 = .0558; *q*_2_ = .0110/.05 = .213; *q*_3_ = .0257/.05 = .507.

The dashed gray lines in the left panel illustrate uniformly distributed conditional *p* values. In case of three studies, these uniformly distributed conditional *p* values should equal ¼, ½, and ¾. Note that the observed conditional *p* values, summing to .0558 + .213 + .507 = .776, are lower than their corresponding expected uniformly distributed conditional *p* values, which sum to ¼ + ½ + ¾ = 1.5. Hence, we see that the conditional *p* values under the null hypothesis (δ = 0) as given in the left side of Figure A2 do not fit a uniform distribution.

To obtain the effect size estimate of *p*-uniform, effect size (δ) has to be shifted until the sum of conditional *p* values equals 1.5, which is the expected value of the sum under uniformity (i.e. given the true effect size). Figure A3 shows the effect of shifting δ on the conditional *p* values from −.5 to 1.5 for the three observed effect sizes in our example. Each conditional *p* value increases when the true effect size gets larger. For instance, the conditional *p* value of Effect 1 increases from .0558 to .25 when the true effect size is increased from 0 to .5, and it is further increased to .459 if true effect size is increased to .748. As a consequence of these increases, the sum of conditional *p* values also increases as true effect size increases.

The middle panel in Figure A2 presents the conditional *p* values in case the effect size is shifted to δ = 0.5. These conditional *p* values are also shown in Figure A3 as the intersections of the three curves with the vertical line representing δ = 0.5 and equal *q*_1_ = .25; *q*_2_ = .50; and *q*_3_ = .75.

These conditional *p* values exactly match (and studies were selected to exactly match) the expected conditional *p* values under uniformity. Consequently, the sum of the conditional *p* values also equals the sum of the conditional *p* values under uniformity (1.5). This indicates that the effect size estimate of *p*-uniform will be equal to the true effect size of 0.5.

The right panel in Figure A2 and the intersections of the curves of the study with line δ = 0.748 in Figure A3 show the conditional *p* values conditional on the effect size δ = 0.748, which was the estimate of traditional fixed-effect meta-analysis:

*q*_1_ = .459; *q*_2_ = .697; *q*_3_ = .868.

All are higher than their corresponding expected conditional *p* values under uniformity, and their sum (2.031) is larger than the expected sum under uniformity (1.5). These results indicate that traditional fixed-effect meta-analysis overestimated the effect size. In such a case, it is not farfetched to suppose that publication bias exists (i.e. some nonsignificant results are missing from the set of studies included in the meta-analysis).

Table A1 shows the results of applying *p*-uniform and *p*-curve to the example. The effect size estimated by *p*-uniform is exactly equal to the true effect size of δ = 0.5. Other results of *p*-uniform are the 95% CI [−0.300, 0.960) and the finding that both the null hypothesis of no effect (*p =* .0737) and the hypothesis of no publication bias (*p* = .147) cannot be rejected.^6^ The output of *p*-curve incorporates neither a confidence interval nor a publication bias test. The *p*-curve estimate of .511 is slightly larger than the true effect size,^7^ and the *p*-curve result of the test of no effect is *p =* .086. Why are the results of both methods different if they are based on the same logic? This is because the methods differ in implementation, which we explain in the Supplemental Materials.

**Table A1.** Results of p-Uniform and p-Curve Applied to the Example Based on Three Observed Effect Sizes

Measurement	*p*-uniform	*p*-curve
Effect size estimate	0.500	0.530
95% CI	[−0.308, 0.964]	
Test of H_0_: δ = 0	*z* = −1.44, *p* = .0753	χ^2^(6) = 1.97, *p* = .0772
Publication bias test	*z* = 1.06, *p* = .144	

*Note*. δ = 0.5. H0: δ = 0 refers to the null hypothesis of no effect. CI = confidence interval.

## Appendix B

**Table B1.** Studies and Corresponding Sample Sizes (Group 1: $n_{i}^{1}$ and Group 2: $n_{i}^{2}$), t Values and Two-Tailed p Values Included in the Meta-Analysis Described in Rabelo, Keller, Pilati, and Wicherts (2015)

Article and experiment	$n_{i}^{1}$	$n_{i}^{2}$	*t*	*p* (two-tailed)
Ackerman, Nocera, and Bargh (2010), Exp. 1	26	28	2.016	.0489
Ackerman et al. (2010), Exp. 2	21	22	1.867	.0690
Chandler, Reinhard, and Schwarz (2012), Exp. 2	30	30	2.554	.0133
Chandler et al. (2012), Exp. 1	50	50	2.113	.0372
Chandler et al. (2012), Exp. 3	50	50	2.390	.0188
Häfner (2013), Exp. 1	30	30	2.042	.0457
Jostmann, Lakens, and Schubert (2009), Exp. 1	20	20	2.245	.0307
Jostmann et al. (2009), Exp. 2	22	28	2.081	.0428
Jostmann et al. (2009), Exp. 3	25	24	2.191	.0335
Jostmann et al. (2009), Exp. 4	20	20	2.294	.0274
Kaspar and Krull (2013)	45	45	3.049	.0030
Kouchaki, Gino, and Jami (2014), Exp. 1a	15	15	2.020	.0531
Kouchaki et al. (2014), Exp. 1c	27	27	2.184	.0335
Kouchaki et al. (2014), Exp. 2	26	25	2.307	.0254
Kouchaki et al. (2014), Exp. 3	35	36	2.308	.0240
Kaspar (2013), Exp. 1	20	20	3.268	.0023
Kaspar (2013), Exp. 2	25.5	25.5	2.306	.0254
Kaspar (2013), Exp. 3	31	31	2.278	.0263
Kaspar (2013), Exp. 4	48.5	48.5	2.053	.0429
Kaspar (2013), Exp. 5	30	30	2.452	.0172
Kouchaki et al. (2014), Exp. 4	31	31	2.139	.0365
Maglio and Trope (2012), Exp. 2	18	18	2.284	.0287
Zhang and Li (2012), Exp. 1	35	35	2.382	.0200
Zhang and Li (2012), Exp. 2	39	39	1.994	.0498
Zhang and Li (2012), Exp. 4	40	40	2.530	.0134

*Note*. Exp. = experiment.

## Notes

1. This principle is one of the most fundamental principles of probability and statistics. For instance, this principle is applied when one is sampling from distributions using so-called *inverse transform sampling* or the *inverse cumulative distribution function method* (Gentle, 2004). In this method, one starts sampling a random number from a uniform distribution from 0 to 1. Next, the random number, which is considered a cumulative percentage of the distribution, is used to calculate the *x* value of the distribution that one wished to sample in the first place.

2. The distribution of (conditional) *p* values based on the true effect size is only uniform when the assumptions of the underlying statistical model (e.g., independence or effect distribution are valid; Bland, 2013]).

3. If *p*-hacking and publication bias were absent in these three studies, the fixed-effect meta-analytic estimator is unbiased and most efficient. In case publication bias was present but none of the three studies involved *p*-hacking, only the estimators of *p*-uniform and *p*-curve would have been accurate. Estimators of both fixed-effect meta-analysis and *p*-uniform and *p*-curve are inaccurate if *p*-hacking was used in the primary studies.

4. Functions for applying *p*-uniform can be loaded in R via the following code: devtools::install_github(“RobbievanAert/puniform”); library(puniform)

5. Due to transformation of *d*_obs_ and *d*_cv_ to *z* values (discussed later in this section), conditional *p* values in *p*-uniform are divided by a value that is slightly larger than .05. Furthermore, dividing by .05 is only feasible if all observed effect sizes are statistically significant in the same direction. Imagine a situation in which the observed effect size of Effect 1 is changed into *d* = −0.872, *t*(48) = −3.133. The two-tailed *p* value of Effect 1 remains .00294, but the observed effect size is statistically significant in the opposite direction from those of Effects 2 and 3. In *p*-uniform and *p*-curve, one-tailed *p* values are used, and consequently, effects with opposite sign will be omitted in *p*-uniform or *p*-curve. If statistically significant effect sizes in a meta-analysis are observed in both tails of the distribution, we recommend applying *p*-uniform and *p*-curve to both the statistically significant observed positive effect sizes and the statistically significant observed negative effect sizes. An example of such an analysis is described in Simonsohn et al. (2014a).

6. The CI in *p*-uniform is obtained via test inversion (e.g., Casella & Berger, 2002), so the lower (upper) bound of the confidence interval equals that effect size for which the sum of conditional *p* values is equal to the 2.5th (97.5th) percentile of the Irwin-Hall distribution. The statistical test of the null hypothesis of no effect of *p*-uniform can be used to examine whether the conditional *p* values follow a uniform distribution if δ = 0 (van Assen et al., 2015). For the publication bias test in *p*-uniform, all studies (significant and nonsignificant) in a meta-analysis are used to compute the effect-size estimate based on fixed-effect meta-analysis. Only the statistically significant studies then are used to examine whether the conditional *p* values follow a uniform distribution conditional on this fixed-effect meta-analytic effect-size estimate. If the statistically significant *p* values are not uniformly distributed conditional on this effect -size estimate, the null hypothesis of no publication bias is rejected (van Assen et al., 2015).

7. For illustrative purposes, we designed an example where the effect-size estimate in *p*-uniform equals the true effect size; an example just as easily can be constructed in which the estimate in *p*-curve equals the true effect size.
